# Something for everyone

**DOI:** 10.7554/eLife.25411

**Published:** 2017-03-15

**Authors:** Sarah Shailes

**Affiliations:** Features, eLife, Cambridge, United Kingdom

**Keywords:** plain-language summaries, scientific publishing, public engagement

## Abstract

Journals and other scientific organizations produce a diverse variety of plain-language summaries.

What do the British Psychology Society, the journal Functional Ecology and an astronomy website called Astrobites have in common? The answer is that they all attempt to explain the findings of research papers to a broad audience by publishing plain-language summaries of the papers. And they are not alone: a growing number of journals and scientific organizations are trying to do the same, and not just for papers on topics that the public are generally thought to be interested in, such as fossils, invisibility cloaks and new medical treatments. These summaries go by a myriad of names – including lay summaries, author summaries, significance statements and digests – and can cover topics as diverse as the discovery of water on a distant planet and the lifestyles of bacteria. To find out more about this phenomenon we contacted ten journals and other organizations that between them produce around 5,400 plain-language summaries per year (Table 1).

**Figure 1:**
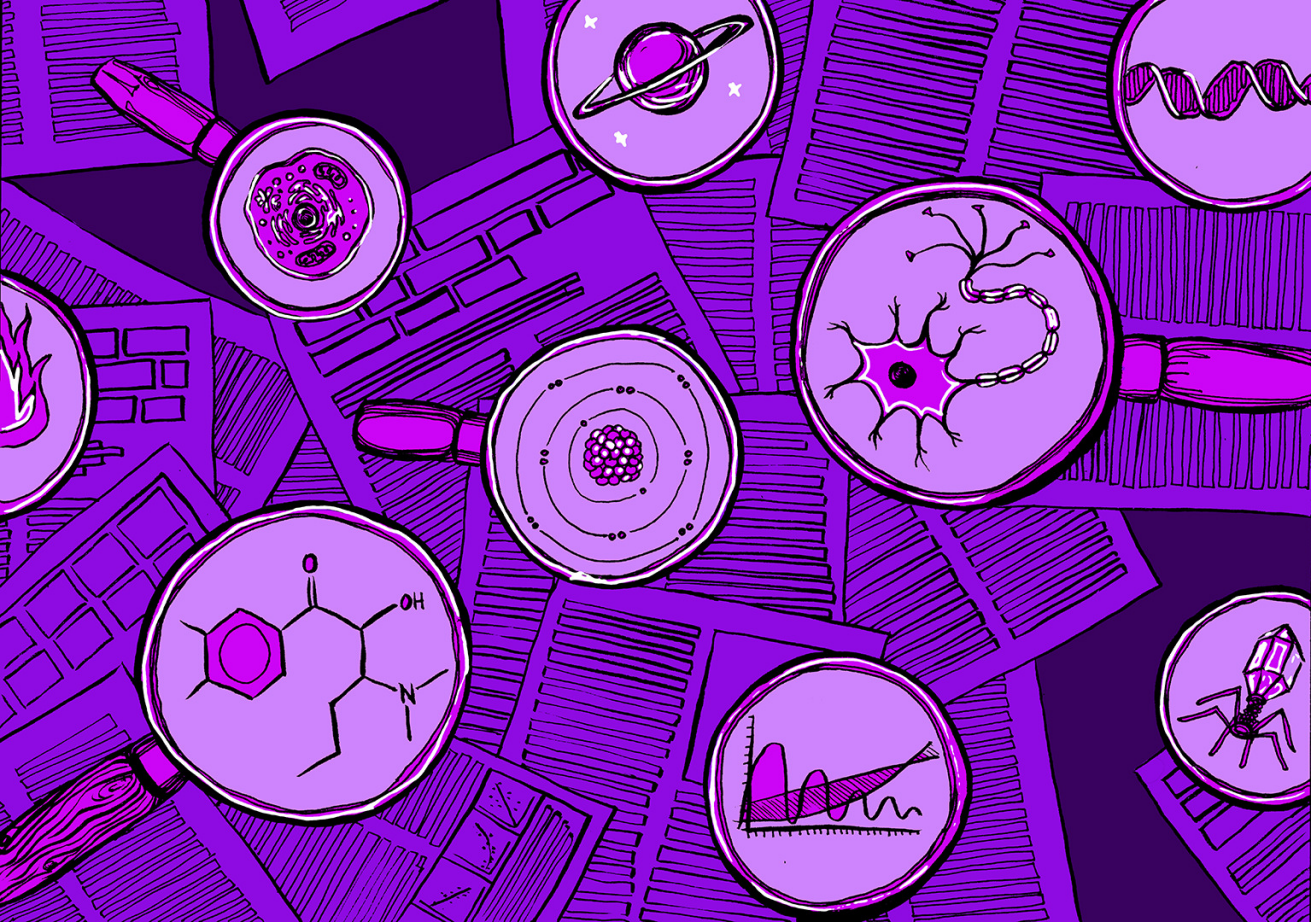
Plain-language summaries are available for research papers in many different areas of science and medicine.

IMAGE CREDIT: vividbiology.com

**Table 1.** A selection of journals, societies and other organizations that produce plain-language summaries of scientific research.

Organization	Type	Name of plain-language summaries	Length	Number of images	Number published per month/year
American Astronomy Society (aasnova.org)	Scientific Society	Highlights	350–500 words	1 image, plus 1–3 figures from article	~12/month
		Journals Digests	One sentence	None	~20/month
Annals of the Rheumatic Diseases (promotions.bmj.com/ardsummaries/)	Journal	Patient summaries	500–1,000 words	None at present	~3/month
Astrobites (astrobites.org)	Student-led organization	N/A	600–800 words	1-2	~20/month
Autism (journals.sagepub.com/home/aut)	Journal	Lay abstracts	250 words max.	None	~80/year
British Psychological Society (digest.bps.org.uk)	Scientific Society	N/A	Variable, generally around 500–700 words	1	~20/month
Cochrane (cochranelibrary.com)	Network of researchers	Plain-language summaries	400–700 words	Usually none	Variable; published 485 in 2015.
eLife (elifesciences.org)	Journal	eLife digests	200–400 words	None	50–60/month
Frontiers for Young Minds (kids.frontiersin.org)	Journal	N/A	2,000 words max.	Variable	Variable; published over 100 articles since launch in 2013.
Functional Ecology (functionalecology.org)	Journal	Lay summaries	250–350 words	1 photograph	~160 articles in 2016
PLOS Medicine (journals.plos.org/plosmedicine/)	Journal	Author summaries	6–9 single sentence bullet points	None	14/month
Proceedings of the National Academy of Sciences (pnas.org)	Journal	Significance Statements	~120 words	None	~3000/year

## Who reads plain-language summaries?

When the British Psychological Society (BPS) started its Research Digest email newsletter back in 2003, its aim was to summarize new psychology research for 16–18 year old school students. "However, we quickly came to realize that we were reaching a much wider audience," says its editor, Christian Jarrett. "For many years now we've been writing for the general public, as well as students, researchers and journalists."

Something similar has happened at Astrobites, a website that publishes plain-language summaries of astrophysics papers on the ArXiv preprint server. At first the site was aimed primarily at undergraduate students studying astrophysics but, again, it has attracted other readers. "Our audience turns out to be approximately equal parts undergraduates, graduate students, professional researchers, and interested members of the broader public," says Nathan Sanders, who helped to launch the site in 2010, when he was a graduate student at Harvard University (Sanders, 2013).

Another Astrobites author, Susanna Kohler, went on to set up AAS Nova for the American Astronomical Society (AAS) in 2015. The website aims to highlight research published in the society’s journals and "provide astrophysics researchers and enthusiasts access to a stream of curated and digested current astrophysics research content". The two initiatives have established a partnership, with some Astrobites summaries appearing on AAS Nova and the AAS helping to fund Astrobites.

Plain-language summaries can help journals in the biomedical sciences to reach out to patients and others who might benefit from the research. The journal Autism has been publishing Lay Abstracts since 2011. The journal's social media editor, Laura Crane, looks after the abstracts. "The target audience is the general public, but particularly the autism community – individuals on the autism spectrum, their families and the people who work with them," says Crane, who is also a researcher at Goldsmiths, University of London.

A major challenge when writing for a broad audience is how to pitch the language, content and style of the text so that it is informative and appealing to people with different levels of scientific education. Many journals and organizations rely on in-house editors to check that their plain-language summaries are suitable for their intended audiences, but some go one step further by including members of their audience in the checking process. At Frontiers for Young Minds (which produces articles aimed at children and teenagers) all articles are reviewed by two people – a young scientist and a research scientist (Box 1).

Box 1. **Opening up science for kids.**

Frontiers for Young Minds is a journal that commissions scientists to write about their research for readers aged between 8 and 15. Authors are given up to 2000 words to translate their work "into terms accessible to kids and teens". A young scientist and a current research scientist then pair up to review the article and provide feedback to the authors. The revised article is then checked by an associate editor, who is an experienced researcher: if this editor is happy with the article they approve it for publication.

Since launching in 2013, the journal has published over 100 articles. "Frontiers for Young Minds flips the scientific process by having kids review scientific publications and as a result, provides an excellent tool for scientists to become better communicators," says Emma Clayton of Frontiers.

The Annals of the Rheumatic Diseases has been producing patient summaries for selected research articles since 2013. "The summaries go through several approval rounds with the patient organization PARE [People with Arthritis and Rheumatism] and the Editor in Chief," says the journal's publishing executive Frances Lee. While this level of checking helps to make the summaries more accessible to patients, it can delay their publication for several months after the research paper is published.

The Cochrane network of medical researchers has been including plain-language summaries in their reviews of the medical literature since 1997. The aim is to "summarize the review in a straightforward style that can be understood by consumers of health care," says Nancy Owens, senior communication manager at Cochrane. Although authors are given guidelines to help them write a summary for their review, a recent study found that none of the plain-language summaries published by Cochrane during a 23-month period fully adhered to these guidelines (Jelicic Kadic et al., 2016). Owens says that Cochrane is currently involved in a project to "to improve and standardize the content, presentation, and readability of plain-language summaries to make them easier to read, use, and translate into other languages."

Some journals and societies produce summaries primarily for scientists working in other fields of research. Since October 2012, all research articles published in the Proceedings of the National Academy of Sciences (PNAS) – which span the biological, physical and social sciences – have included a Significance Statement to explain the relevance of the work to the readers of the journal, as opposed to the general reader (Verma, 2012).

And last year Nature commissioned the authors of 11 articles in the journal to write summaries of their research papers. Again these summaries – which ran to two A4 pages in pdf – were primarily aimed at other researchers rather than the public: "The summaries remain technical," explained an editorial in the journal, "these are not articles suitable for the popular press" (Nature, 2016). "We are still looking at the feedback from the trial," says Alice Henchley, communications director for Nature Research, "but is clear that the summaries were well received."

## Who should write the summary?

Most of the journals published by Public Library of Science (PLOS) – including PLOS Biology, PLOS Genetics and PLOS Medicine – include plain-language summaries in all of their research articles. When PLOS Medicine launched in 2004, it employed freelance writers to prepare the summaries for the articles. Last year, however, the journal changed its approach and started to ask authors to draft their own. Chief Editor Larry Peiperl explains: "We thought it would make for a more efficient process – author drafts, editor edits and author approves, rather than, in the most extended case, freelancer drafts, editor checks, author reviews and makes edits, editor approves or revises further – and better use of resources." The journal was initially worried about how authors would respond to the new approach, but the change appears to have been accepted. "The transition went remarkably smoothly," says Peiperl. "The authors seem quite willing, and generally we’ve been happy with the quality and clarity of their summaries."

Like PLOS Medicine, many other journals – including Autism and Functional Ecology – also ask the authors of research articles to write their own plain-language summaries. Aware that the authors may not have much experience of writing for a broad audience, the journals check and, if necessary, edit the summaries before publication.

Some journals and other organizations employ writers to produce plain-language summaries. To help their writers, Annals of the Rheumatic Diseases and eLife both ask the authors of research papers to answer a set of questions about their work in plain language. At eLife we have found that involving the original authors in this way can save time later because they tend to make fewer changes when they check the draft summary.

When is a good time to ask authors to draft a summary? PNAS require authors to include a Significance Statement when they first submit an article to the journal. However, if the paper is rejected and eventually published without the statement in another journal, the authors’ efforts will have been wasted. Autism avoids this problem by asking authors to produce a Lay Abstract once their paper has been accepted for publication. Functional Ecology and PLOS Medicine, on the other hand, ask authors to draft a plain-language summary when they are revising their article after peer review: this gives the journal and the authors time to check and revise the summary before the article is published.

Astrobites employs a different model to write its summaries. "All our authors are current astronomy graduate students at universities worldwide who volunteer," says Nathan Sanders. "We have a 'regular rotation' of about 20 authors at a time so that each author writes one post per month." They use a 'collaborative authorship model' in which every author also acts as a content editor for another student in the collaboration and as a style editor for someone else. In this way, Astrobites is able to coordinate the efforts of a team of volunteers to produce a regular stream of content.

Their collaborative authorship model has proved popular in other disciplines and Astrobites now has sister sites covering oceanography (oceanbites.org), particle physics (particlebites.com) and astronomy in Spanish (astrobitos.org). It is also re-launching a site for chemistry (chembites.org). Alongside its summaries of research, Astrobites also publishes content about careers and other issues. In 2013, to help young scientists develop their science communication skills, some of the authors from Astrobites and Chembites also founded ComSciCon, which runs workshops for graduate students in the US.

Astrobites and its sister sites are not alone in providing researchers with opportunities to write plain-language summaries for research carried out by other scientists. Annals of Botany, The Node and various other organizations also publish plain-language summaries written by volunteers, generally in the form of blog posts.

## Where to find them

Different organizations also display their plain-language summaries in different ways. Some journals – including PLOS Medicine, PNAS and eLife – display the summary within the original research article. While this might be convenient for scientists and other readers who want to get a general overview of the article before they read more, it could deter or prevent many non-scientists from ever reaching the article in the first place.

Functional Ecology – which has published plain-language summaries for all research articles since 2011 – gets around this problem by presenting the summaries as separate articles with images for visual interest. The summaries of recent articles are highlighted on the journal’s homepage like news pieces. This approach also allows Functional Ecology to make all of its plain-language summaries freely available, even though many of the related research articles are not open access. The Annals of the Rheumatic Diseases goes a step further by displaying its patient summaries in a dedicated section of its website: this section has a separate search index that allows patients and other members of the public to search the summaries using keywords.

Another way to make plain-language summaries easier to find is to display them in more than one place. Following the success of the Research Digest newsletter, the BPS launched its blog in 2005 "as a way to give the Digest more of a magazine style with images and the chance for readers to comment," says Christian Jarrett. Along with the newsletter and blog, the BPS developed an app last year to help readers find and manage Research Digest content more easily.

While researching this article we compiled a list of 50 journals that produce plain-language summaries, with recent additions to the list including JGR: Planets (Hansen, 2016) and Evolution (Noor, 2017). (The full list is available in this blogpost). These journals and other scientific organizations are taking a variety of approaches to writing and displaying their summaries, with different target audiences in mind. This diversity should mean that there will be something out there for everyone interested in science, regardless of their scientific background, providing they know where to look.
